# High mortality in North American Natives with systemic lupus erythematosus

**DOI:** 10.1186/ar4657

**Published:** 2014-09-18

**Authors:** Christine A Peschken, Ripneet Puar, Carol A Hitchon

**Affiliations:** 1 University of Manitoba, Winnipeg, MB, Canada

## Background

Lupus outcomes including mortality have been found to be worse in most ethnic minorities, but little is known about North American Indigenous people (NAI). We compared mortality in NAI systemic lupus erythematosus (SLE) patients with Caucasian and other ethnic minority (OEM) SLE patients at a single academic center.

## Methods

Patients were followed from 1990 to 2013 using a custom database. Variables included date of birth, diagnosis, year of disease onset, ethnicity, clinic visit dates, and vital status if known. Records of all patients with a diagnosis of SLE (≥4 American College of Rheumatology criteria) were abstracted. For patients who had not been seen in the last 2 years, updated vital status was obtained from the hospital medical records department. Ethnicity was by self-report, and categorized into NAI, Caucasian and OEM. The age at diagnosis, disease duration and age at last follow-up or age at death was calculated and compared between ethnic groups. Survival time was compared between ethnic groups using Kaplan-Meier and Cox proportional hazard models.

## Results

**Figure 1:**
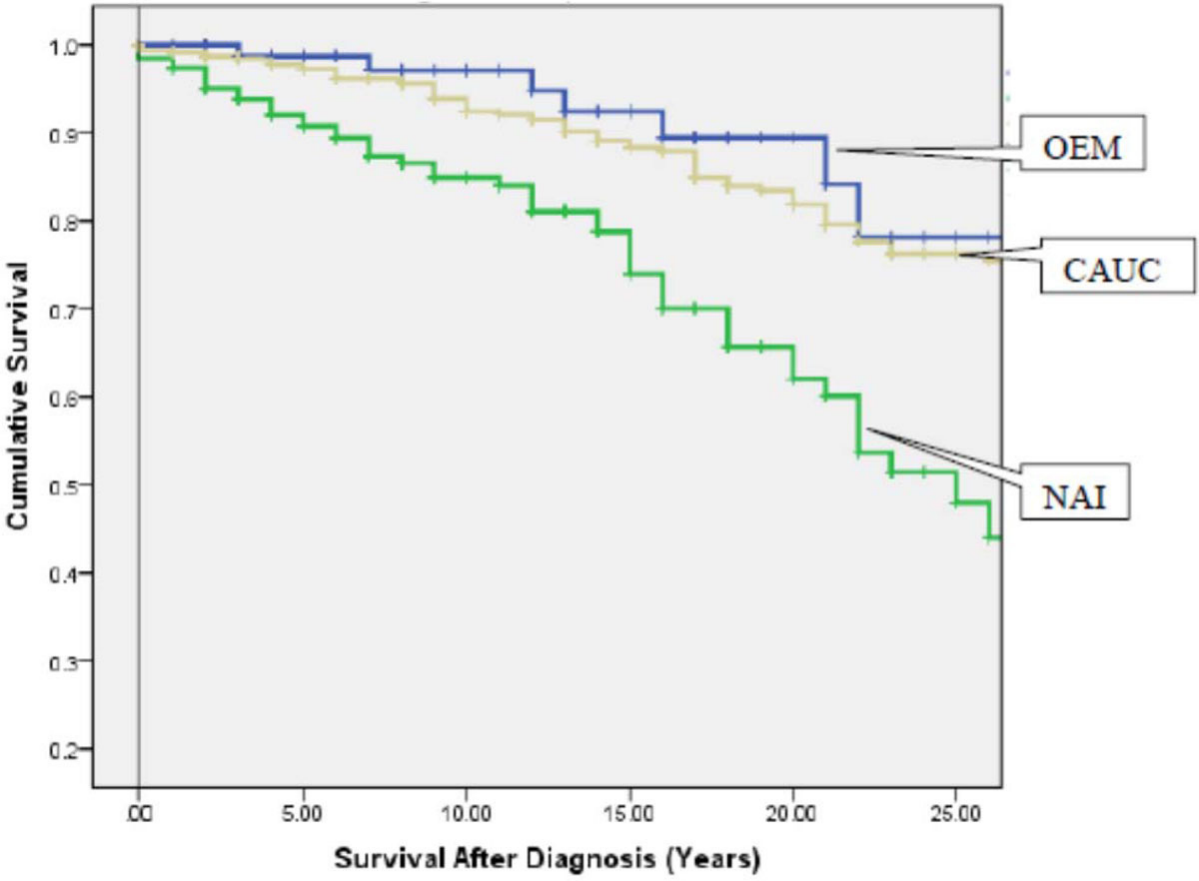
**Kaplan-Meier survival curve**.

A total of 807 patients with SLE were identified: 201 (25%) patients were NAI, 501 (62%) were Caucasian, and the remaining 105 (13%) were OEM. NAI and OEM patients were younger at diagnosis (NAI = 32 ± 15 years; OEM = 31 ± 14 years; Caucasian = 37 ± 15 years; *P *= 0.001), had a shorter disease duration (NAI = 11 ± 9 years; OEM = 10 ± 9 years; Caucasian = 15 ± 11 years; *P *= 0.001) and had more frequent nephritis (NAI = 41%; OEM = 49%; Caucasian = 29%; *P *= 0.001) compared with Caucasians. More NAI had died by the end of the follow-up period (NAI = 25%; OEM = 7%; Caucasian = 18%; *P *< 0.001) and mean age at death was much younger in both NAI and OEM (NAI = 50 ± 16 years; OEM = 46 ± 14 years; Caucasian = 63 ± 16 years; *P *= 0.001). Survival rates were significantly worse in NAI compared with OEM and Caucasians (Figure 1): 10-year survival 85% versus 97% and 92%; 15-year survival 75% versus 89% and 88% respectively (*P *< 0.001). In a Cox proportional hazards model, the risk of death following diagnosis was higher for NAI (hazard ratio 2.5; 95% CI: 1.6 to 3.9) after adjustment for onset age, damage, and lupus manifestations.

## Conclusions

NAI and OEM patients had similarly young onset age and more frequent nephritis, but survival was markedly worse in NAI compared with Caucasians. Urgent improvements in care delivery for NAI with SLE are needed to decrease the significant morbidity and mortality burden from this disease.

